# Delipidation of Plasma Has Minimal Effects on Human Butyrylcholinesterase

**DOI:** 10.3389/fphar.2018.00117

**Published:** 2018-02-15

**Authors:** Seda Onder, Ozden Tacal, Oksana Lockridge

**Affiliations:** ^1^Department of Biochemistry, School of Pharmacy, Hacettepe University, Ankara, Turkey; ^2^Eppley Institute, University of Nebraska Medical Center, Omaha, NE, United States

**Keywords:** Aerosil 380F, dextran sulfate, fatty plasma, butyrylcholinesterase, delipidation, monoclonal antibodies

## Abstract

Human butyrylcholinesterase (BChE) is purified in large quantities from Cohn fraction IV-4 to use for protection against the toxicity of chemical warfare agents. Small scale preliminary experiments use outdated plasma from the American Red Cross as the starting material for purifying BChE (P06276). Many of the volunteer donor plasma samples are turbid with fat, the donor having eaten fatty food before the blood draw. The turbid fat interferes with enzyme assays performed in the spectrophotometer and with column chromatography. Our goal was to find a method to remove fat from plasma without loss of BChE activity. Satisfactory delipidation was achieved by adding a solution of 10% dextran sulfate and calcium chloride to fatty plasma, followed by centrifugation, and filtration through a 0.8 μm filter. Treatment with Aerosil also delipidated fatty plasma, but was accompanied by loss of 50% of the plasma volume. BChE activity and the BChE isozyme pattern on nondenaturing gel electrophoresis were unaffected by delipidation. BChE in delipidated plasma was efficiently captured by immobilized monoclonal antibodies B2 18-5 and mAb2. The immunopurified BChE was released from antibody binding with acid and visualized as a highly enriched, denatured BChE preparation by SDS gel electrophoresis. In conclusion, delipidation with dextran sulfate/CaCl_2_ preserves BChE activity and the tetramer structure of BChE.

## Introduction

Lipemia in blood is caused by a rise in chylomicron particles following a meal containing fat. Within 1 h after a meal, fat is evident in human blood and continues to rise, peaking between 2 and 4 h (Gage and Fish, 1924). If no additional food is consumed, the fat is cleared to baseline levels after 8 to 10 h. Patients scheduled for blood tests are asked to fast overnight because lipemic blood can interfere with clinical biochemical tests for alanine aminotransferase, total protein, phosphorus, creatinine, and calcium concentration (Calmarza and Cordero, 2011). Lipemic blood has been reported to interfere with electrophoretic analysis of alpha-2-globulin. Lipoproteins can interfere with clinical serology tests by blocking binding sites on antibodies (Nikolac, 2014).

Volunteers who donate blood to the American Red Cross are encouraged to eat before they donate blood. A significant number of bags of outdated plasma that we receive for research are opalescent with fat. We purify butyrylcholinesterase from plasma by column chromatography and find that fatty plasma fouls our chromatography media. We investigated methods for delipidating plasma so that we would be prepared to process large volumes of human plasma through our BChE purification protocol.

Human plasma containing genetic variants of BChE may be lipemic (Delacour et al., 2014a,b). The spectrophotometric assays we use require pipetting 10 or 20 μL plasma. Fat in plasma coats the pipette tip inside and out, creating uncertainty in the volume of plasma delivered and inconsistency in assay results. Our goal was to identify a process that yields the highest volume of delipidated plasma and to determine whether the delipidation reagent affects BChE activity, binding to antibodies, performance on affinity chromatography, and visualization of BChE isozymes on denaturing gel electrophoresis.

Lipids can be extracted from plasma or serum with chloroform/methanol or hexane/isopropanol (Ferraz et al., 2004). Treatment with organic solvents can destroy enzyme activity. For example, extraction of human serum with butanol-diisopropyl ether is accompanied by loss of 80 to 90% of alkaline phosphatase and lactate dehydrogenase activity (Agnese et al., 1983). BChE is inactivated by 25% ethanol and 3% n-butyl alcohol (Whittaker, 1968). Methanol changes the kinetic properties of BChE (Ferro and Masson, 1987). To preserve BChE activity we avoiding using organic solvents to delipidate plasma and instead tested solid phase extraction reagents.

## Materials and methods

Outdated, once-frozen and thawed human plasma in citrate phosphate dextrose anticoagulant was obtained from the University of Nebraska Hospital Blood Bank. The plasma had no identifiers, thus classifying use of the plasma as exempt from regulations pertaining to human specimens, according to United States law 45 CFR 46.101(b). The volunteer donor blood had been collected and tested for pathogens by the American Red Cross. Dextran sulfate (Sigma D-6001), Aerosil 380F, acid washed (Univar). Hupresin affinity gel was synthesized by Emilie David (CHEMFORASE, Mont-Saint-Aignan, France).

### Delipidation of fatty plasma with calcium chloride and dextran sulfate

Fatty plasma was identified by its cloudy appearance. The presence of fat was confirmed by centrifuging a 1 mL aliquot at 4°C and finding a layer of fat on the surface. Donors who ate a high-fat meal before blood draw have lipemic blood.

Two methods for delipidation using dextran sulfate and calcium chloride were tested. The initial strategy followed the method of Proksch and Bonderman (1981). One Liter of plasma was adjusted to 50 mM CaCl_2_ by adding 20 mL of 2.5 M CaCl_2_. Dextran sulfate (0.3 g) was hydrated in 40 mL plasma on a rotating mixer for 1 h at room temperature. The hydrated dextran sulfate was added to plasma in small aliquots while mixing the plasma. After overnight storage at 4°C the treated plasma was centrifuged at 14,000 rpm (23,000 × g) in an SS34 rotor in a Sorvall RC5C refrigerated centrifuge for 30 min at 4°C. A cream-colored pellet was discarded. The supernatant was hazy but free of fat. Storage at 4°C for 2 days or more caused a white precipitate to settle out, leaving a clear delipidated plasma solution. Slow addition of the 40 mL of dextran sulfate was critical. Rapid addition resulted in immediate formation of a gelatinous lump occupying 400 mL. The gelatinous lump only partly dispersed during stirring and did not collapse during centrifugation.

The second strategy was that of Masseyeff et al. (1965). A 10% (w/v) solution of dextran sulfate was prepared by hydrating 0.5 g dextran sulfate in 5 mL water. This made a clear solution. A 10 mL aliquot of fatty plasma was treated with 0.1 mL of 10% dextran sulfate and 0.2 mL of 2.5 M CaCl_2_. The suspension was rotated for 2 h at room temperature. Centrifugation at 10,000 rpm for 30 min at 4°C yielded delipidated plasma. Dextran sulfate particles settled out over a period of days. Alternatively, the delipidated plasma was filtered through a 0.8 μ filter to remove residual dextran sulfate. The Masseyeff approach was preferred over that of Proksch and Bonderman because it is simpler. Note that when solid dextran sulfate was added directly to plasma without first being hydrated, the fatty plasma remained turbid.

### Delipidation with aerosil 380F

Use of Aerosil 380 to delipidate human serum is described by Agnese et al. (1983). Aerosil 380F is a fluffy white substance that occupies 5.5 mL for 0.2 g dry weight. Aerosil (0.2 g) was added to a 15 mL plastic tube in a fume hood to avoid inhaling the silica particles. Fatty plasma (10 mL) was added and the tube rotated at room temperature for 2 h. Centrifugation at 4,000 rpm in a table-top Sorvall centrifuge brought down a pellet that occupied 2 mL. There was a layer of fat on top of the plasma. An additional 0.3 g of Aerosil was added to the tube. After 2 h rotation the Aerosil had swollen to 10 times its weight. Centrifugation resulted in 5 mL of solid and 5 mL of delipidated plasma.

### Enzyme activity assay

BChE activity was measured in 0.1 M potassium phosphate pH 7.0 with 1 mM butyrylthiocholine iodide and 0.5 mM 5,5′-dithiobis(2-nitrobenzoic acid) (Sigma D1830). The rate of increase in absorbance at 412 nm was converted to μmoles hydrolyzed per min using the extinction coefficient E_412nm_ = 13,600 M^−1^ cm^−1^ (Ellman et al., 1961). A unit of activity is defined as 1 μmoles hydrolyzed per minute.

BChE activity was visualized with the above reagents on beads carrying the BChE-antibody complex. Following addition of the reagents the beads turned bright yellow when BChE was in the complex.

### Polyacrylamide slab gel electrophoresis

Polyacrylamide gels were poured in-house in a Hoefer Scientific SE600 vertical electrophoresis system. The separating gel contained a 4–30% gradient of acrylamide. The stacking gel was 4% acrylamide. Nondenaturing gels were electrophoresed at 320 volts constant voltage at 4°C for 20 h and stained for BChE activity by the method of Karnovsky and Roots using butyrylthiocholine iodide as substrate (Karnovsky and Roots, 1964). SDS gels were electrophoresed at 150 v for 16 h at room temperature and stained with Coomassie blue.

### Affinity chromatography

Pharmacia C10/10 column was packed with 2 mL Hupresin affinity gel. The gel was equilibrated with 20 mM TrisCl pH 7.5, 0.05% azide. Human plasma (100 mL) delipidated with dextran sulfate and 50 mM calcium chloride was pumped onto the column at a flow rate of 0.2 mL/min. The gel was washed with 20 mM TrisCl pH 7.5, 0.05% azide (22 mL) followed by 0.3 M NaCl in buffer (22 mL). BChE was eluted with 10 mL of 0.1 M tetramethylammonium bromide in 20 mM TrisCl pH 7.5, 0.05% azide.

### Antibody binding

Mouse anti-human BChE monoclonals B2 18-5 (accession KT189143 and KT189144) and mAb2 (accession KJ141199 and KJ141200) were prepared as described (Peng et al., 2015). The monoclonals were immobilized on Sepharose beads and stored in phosphate buffered saline containing 0.1% sodium azide. A 0.1 mL suspension contained 33 μg antibody bound to 20 μL beads. Eight microfuge tubes each received 0.1 mL suspension of immobilized B2 18-5 and eight received 0.1 mL suspension of immobilized mAb2. After brief centrifugation in a microfuge, the liquid from 0.1 mL suspension was discarded. Duplicate 1.5 mL samples of control plasma (minimal fat), fatty plasma, dextran sulfate treated plasma, and Aerosil 380F treated plasma were added to each set of B2 18-5 and mAb2 beads. Tubes were rotated overnight at room temperature. BChE activity was tested in the unbound supernatant plasma and compared to activity in plasma before incubation with immobilized antibody.

### Visualization of BChE bound to immobilized monoclonal antibodies

To confirm that the BChE in plasma had been captured by immobilized antibodies rather than simply inactivated, we extracted bound protein from the beads and visualized the extracted proteins on an SDS slab gel stained with Coomassie blue. In brief, beads were transferred to 0.45 micron spin filters and washed with 0.5 mL aliquots of phosphate buffered saline until the absorbance at 280 nm of the flow through was <0.04. The washed beads were desalted by two washes with 0.5 mL water. Bound proteins were released from the antibody by two treatments with 50 μL of 1% trifluoroacetic acid. The extracts were dried in a vacuum centrifuge, dissolved in 20 μL of SDS/dithiothreitol, glycerol, bromphenol blue, denatured in a boiling water bath for 3 min and loaded on an SDS gel.

## Results

### Delipidation with dextran sulfate and calcium chloride

**Figure 1:**
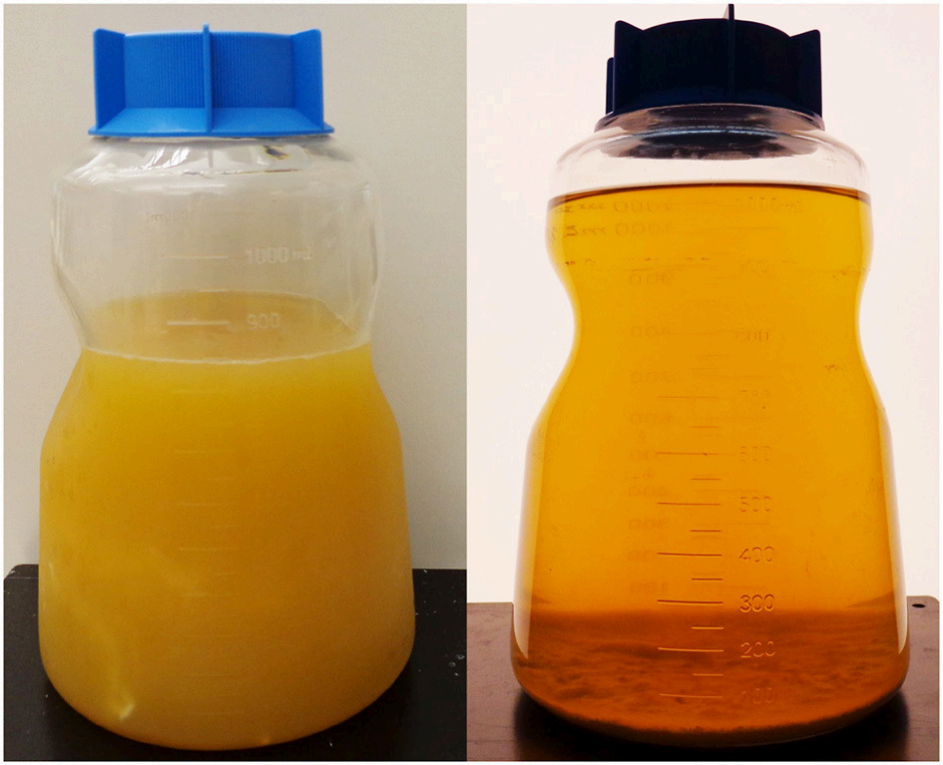
Fatty plasma before and after delipidation with dextran sulfate and calcium chloride.

The photograph in Figure 1 illustrates the difference in turbidity of a 1 L plasma sample before and after delipidation with dextran sulfate and calcium chloride according to the method of Proksch and Bonderman et al. (Proksch and Bonderman, 1981). Centrifugation of the plasma following the initial treatment yielded a hazy solution that clarified during storage at 4°C for 2 days or more, the particles having settled to the bottom of the bottle. After delipidation, the plasma was optically clear. The same result was obtained from the method of Masseyeff et al. (Masseyeff et al., 1965) but the Masseyeff method was simpler and therefore preferred.

### Delipidation with aerosil 380F

**Figure 2:**
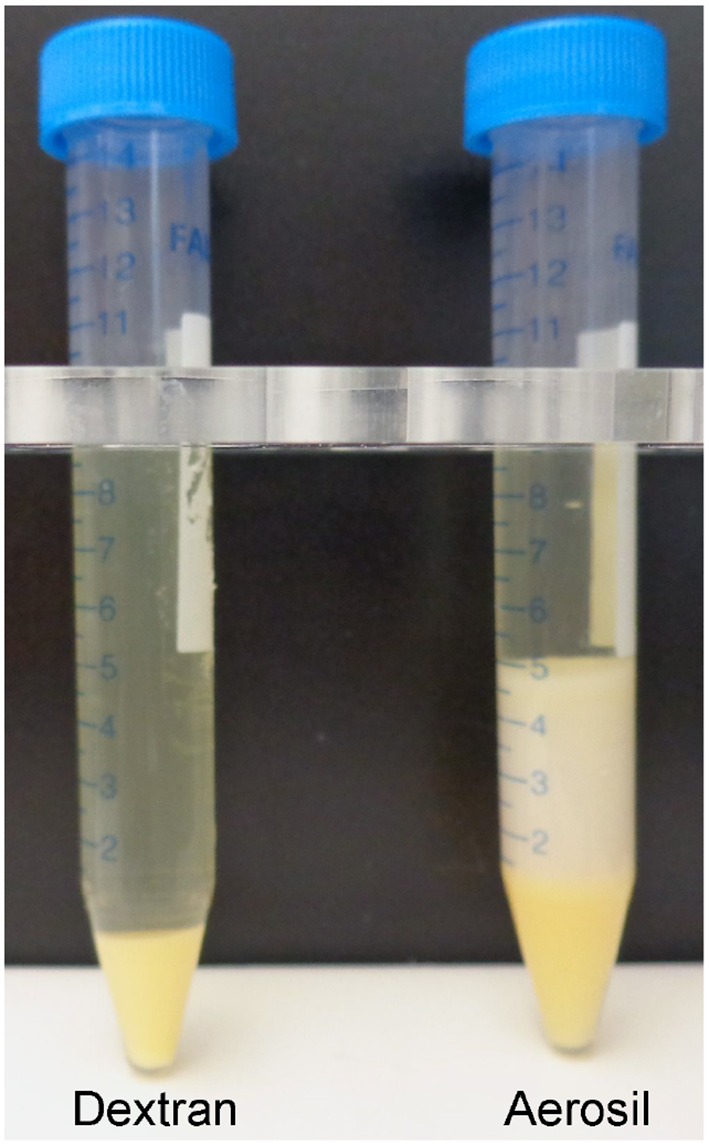
Human plasma delipidated with dextran sulfate/CaCl_2_ or Aerosil 380F. The 10 mL of fatty plasma yielded 9.2 mL of delipidated plasma after treatment with dextran sulfate and CaCl_2_ (Masseyeff et al., 1965) and 0.8 mL pellet. The Aerosil treated plasma yielded 5 mL of delipidated plasma and 5 mL pellet. The Aerosil pellet has 2 colors. The darker yellow on the bottom is from the first attempt to delipidate with 0.2 g Aerosil. The lighter color on the top is from the second attempt with an additional 0.3 g Aerosil.

Fatty plasma treated with acid washed Aerosil 380F was successfully delipidated, but lost half of the plasma volume to the Aerosil pellet. 0.2 g of Aerosil in 10 mL plasma resulted in partial delipidation. Addition of a total of 0.5 g of Aerosil was required for complete delipidation. The 0.5 g of solid Aerosil swelled to 5 g and consumed 5 mL of the original 10 mL plasma (see Figure 2, Table 1).

**Table 1:** Effect of delipidation on BChE activity and plasma volume.

**Method**	**u/mL before**	**u/mL after**	**Volume before**	**Volume after**
Dextran sulfate/CaCl_2_	1.8 ± 0.1	1.9 ± 0.1	10 mL	9.2 mL
Aerosil 380F	1.8 ± 0.1	1.9 ± 0.1	10 mL	5 mL

*Activity for triplicate assays is shown as the mean ± standard deviation*.

### BChE activity and volume in delipidated plasma

Plasma delipidated with dextran sulfate/ CaCl_2_ or with Aerosil 380F had the same BChE activity per mL before and after treatment. As indicated in Table 1 and illustrated in Figure 2, a major difference between the delipidation processes was the yield of delipidated plasma. Delipidation with dextran sulfate/CaCl_2_ yielded 92% of the original plasma volume, whereas delipidation with Aerosil yielded 50%. For experiments requiring the maximum recovery of plasma, delipidation with dextran sulfate/calcium chloride was preferred.

### Nondenaturing gel electrophoresis of fatty and delipidated plasma

**Figure 3:**
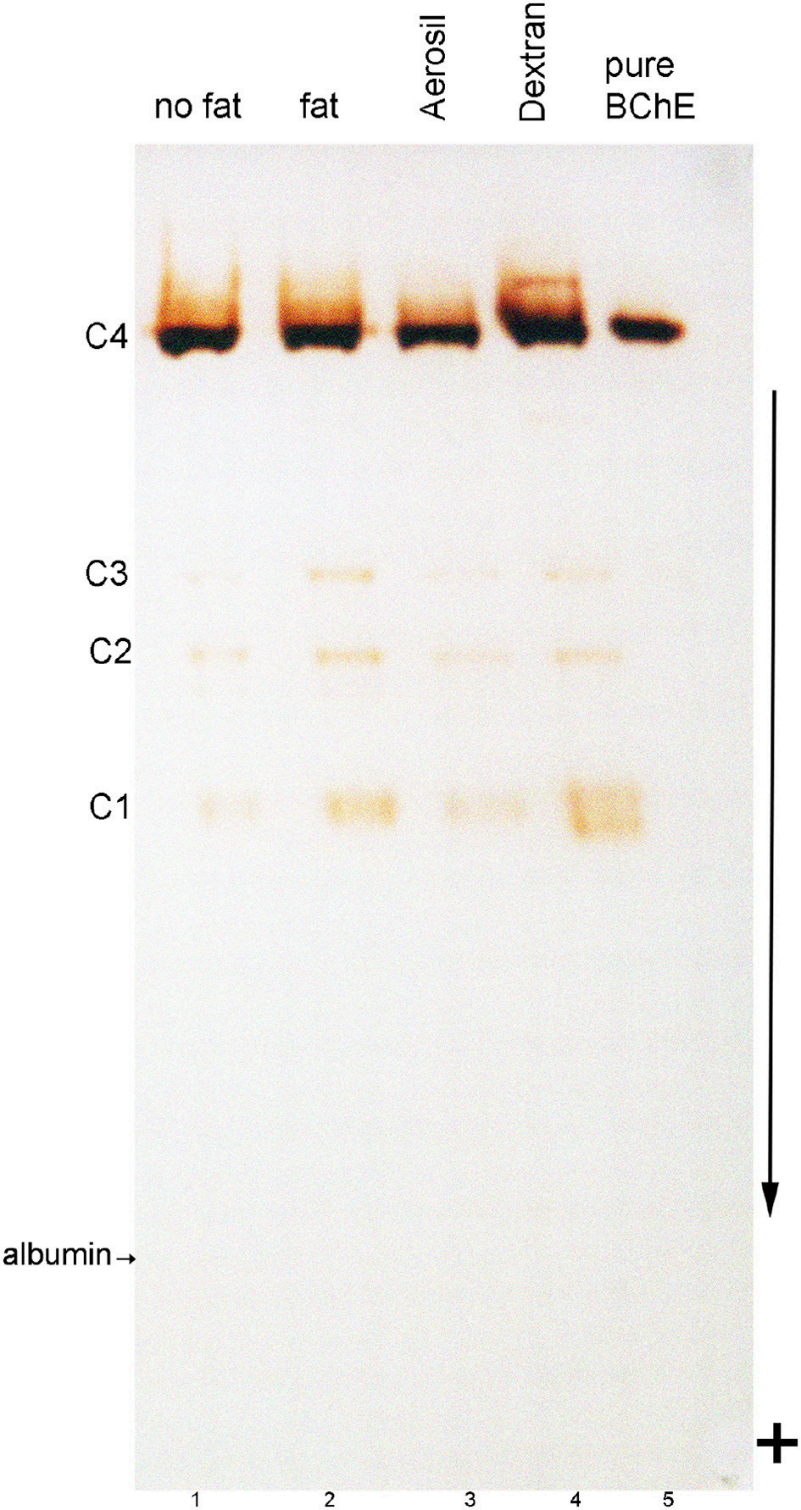
Nondenaturing gel stained for BChE activity. Lanes were loaded with 3 μL plasma. Lane 1, control plasma with minimal fat; lane 2, fatty plasma; lane 3, fatty plasma delipidated with Aerosil 380F; lane 4, fatty plasma delipidated with dextran sulfate/CaCl_2_; Lane 5 contains 0.005 units of BChE (0.01 μg) purified from Cohn fraction IV-4. Bands of BChE activity labeled C1, C3, and C4 are BChE monomer, dimer, and tetramer. The C2 band is a dimer containing one molecule of BChE and one molecule of albumin (Masson, 1989). The location of albumin was detected by counterstaining the gel with Coomassie blue. Albumin separates from BChE on a nondenaturing gel. The arrow indicates the direction of protein migration; sample wells are at the top of the gel.

The nondenaturing PAGE gel of plasma stained for BChE activity in Figure 3 shows the C4 tetramer band of BChE and the minor BChE isozymes C1, C2, and C3 in all samples (lanes 1–4). In contrast, pure BChE purified by ion exchange and affinity chromatography on Hupresin (lane 5) consists exclusively of tetramers. Both control plasma, having no visible fat, (lane 1) and fatty plasma (lane 2) have an indistinguishable pattern of BChE bands. All plasma samples have a faint fuzzy region above the C4 band. This fuzzy region is broader in the dextran treated sample (lane 4), and is absent in pure BChE (lane 5). The dextran treated plasma also has a broader C1 band (lane 4). The fact that delipidated plasma consists predominantly of C4 means the delipidating agents did not disrupt the tetrameric structure of BChE.

### Residual dextran particles on hupresin affinity chromatography

**Figure 4:**
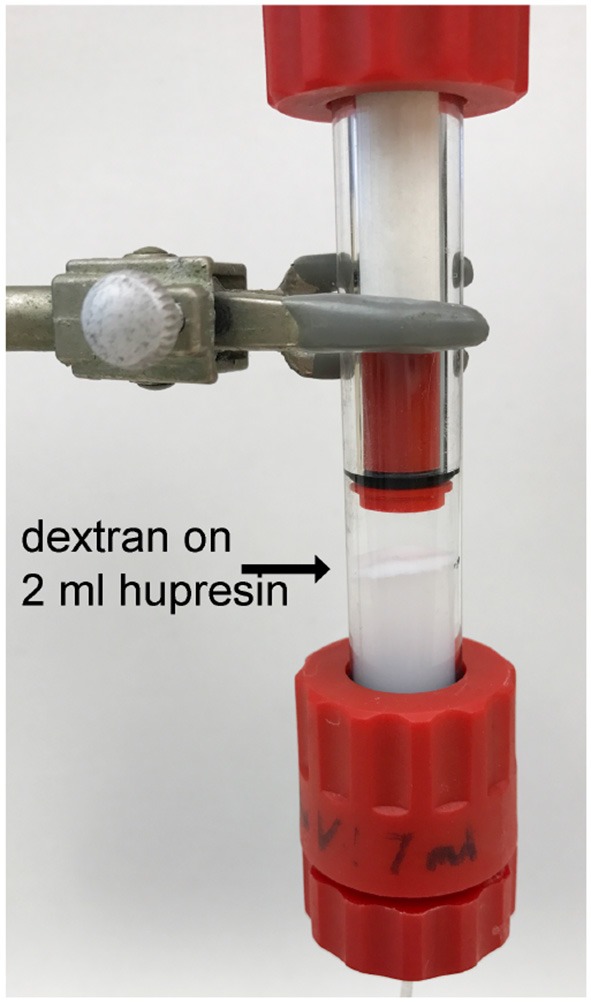
Hupresin affinity gel (2 mL) loaded with 100 mL delipidated plasma deposited a white layer of dextran on the Hupresin surface.

Plasma delipidated with dextran sulfate/CaCl_2_ was clarified by centrifugation followed by settling to produce the optically clear plasma in Figure 1. A 100 mL aliquot of the clarified plasma was pumped onto a 2 mL column of Hupresin. A white layer of dextran sulfate was deposited on top of the Hupresin. Figure 4 shows that the dextran layer was no more than 2 mm thick. The binding capacity of 2 mL Hupresin was reduced for the dextran delipidated plasma from 85 to 90% for control plasma to 78% for dextran plasma. The chromatography was repeated after the delipidated and clarified plasma was filtered through a 0.8 μ vacuum filter. The 100 mL of filtered delipidated plasma left no residue on top of 2 mL Hupresin.

### Minimal interference with antibody binding

The first goal was to determine how fat and delipidating agents affected BChE binding to immobilized antibodies. BChE activity was measured in plasma before and after incubation with immobilized monoclonals B2 18-5 and mAb2. The residual unbound activity was used to calculate percent bound. Table 2 shows that the presence of fat in plasma slightly reduced BChE binding to antibodies from 98% for control to 95–96% for fatty plasma. The dextran sulfate delipidated plasma had slightly reduced binding to mAb2, 91% of the activity in 1.5 mL plasma being captured by mAb2, whereas binding to B2 18-5 was unaffected. Delipidation with Aerosil 380F did not interfere with BChE binding to either immobilized monoclonal.

**Table 2:** Binding capacity of immobilized monoclonals for fatty and delipidated plasma.

**Plasma**	**% BChE bound to B2 18-5**	**% BChE bound to mAb2**
Control	98 ± 1	98 ± 1
Fatty	95 ± 2	96 ± 2
Dextran-treated	97 ± 1	91 ± 1
Aerosil-treated	98 ± 1	98 ± 1

% bound was calculated from triplicate activity assays. Statistical calculation show the mean ± standard deviation

**Figure 5:**
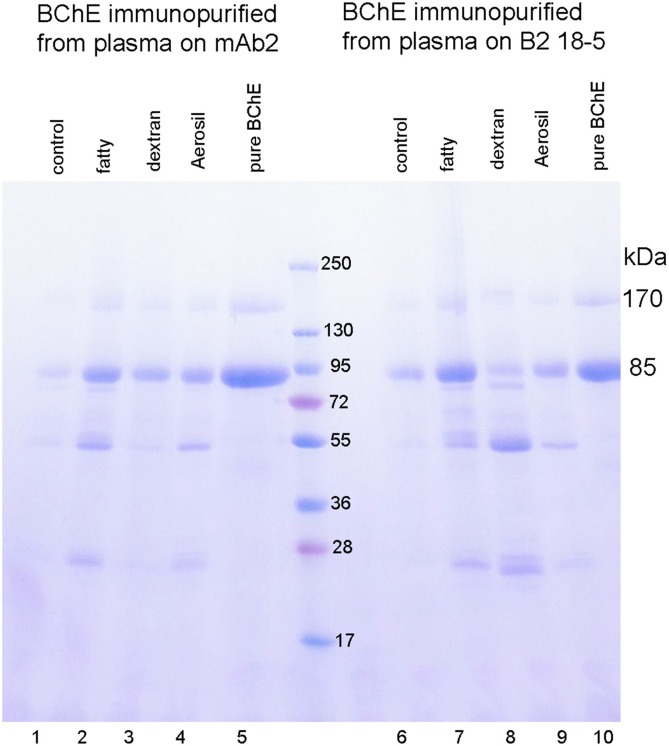
BChE immunopurified from 1.5 mL plasma by binding to immobilized monoclonal mAb2 or B2 18-5. BChE was dissociated from the washed beads with 1% trifluoroacetic acid. The Coomassie stained SDS PAGE gel shows a monomer band for pure BChE at 85 kDa and a dimer band at 170 kDa in lanes 5 and 10. The BChE protein captured by mAb2 in lanes 1–4, and by B2 18-5 in lanes 5–9 has an intense band at 85 kDa for the BChE monomer and a band at 170 kDa for the BChE dimer. The immunopurified BChE has contaminating bands at 55 and 25 kDa. Differences in band intensities are explained by handling losses of 20 μL beads.

Our second goal was to certify that the disappearance of BChE activity from plasma following incubation with immobilized monoclonals was due to capture of the BChE by the antibodies and not to inactivation. Two methods provided evidence that BChE had been captured by the immobilized antibodies. In the first method an aliquot of the reagents that measure BChE activity in the spectrophotometer was added to the washed BChE-antibody beads. The beads and the solution turned bright yellow immediately on addition of the reagents, evidence for the presence of active BChE on the beads. The second method required denaturation of the BChE protein to release BChE from the antibody complex. We have not found a method to dissociate native BChE from the BChE-antibody complex. Addition of 1% trifluoroacetic acid to the washed beads, dissociated bound proteins from the antibodies. The released proteins were visualized on the SDS PAGE gel in Figure 5. Lanes 1–4 show the proteins captured from plasma by mAb2 and released with acid. Lanes 6–9 show the proteins captured from plasma by B2 18-5 and released with acid. The BChE monomer, at 85 kDa, is present in all samples and is the most intense band. A nonreducible BChE dimer is present at 170 kDa. Contaminating proteins are present at 55 and 25 kDa. Band intensities differ in the samples but these differences do not reflect the binding capacity of the monoclonals. The differences are explained by handling losses for the very small 20 μL volume of beads.

The gel in Figure 5 shows the remarkable level of BChE enrichment achieved in a single step by treating plasma with immobilized monoclonals mAb2 and B2 18-5. Plasma contains only 4 μg BChE per mL and 60,000 μg/mL albumin, IgG and other proteins. Figure 5 demonstrates that the antibodies are highly specific for BChE. It was concluded that monoclonals mAb2 and B2 18-5 immobilized on Sepharose beads have high selectivity and high binding capacity for BChE in plasma and that fat and delipidating agents have minimal effects on the ability of immobilized antibodies to capture BChE from plasma.

## Discussion

We used established protocols to delipidate plasma (Masseyeff et al., 1965; Proksch and Bonderman, 1981; Agnese et al., 1983). Our goal was to identify the protocol that best fit our work with human butyrylcholinesterase. We concluded that delipidation with dextran sulfate/CaCl_2_ using the method of Masseyeff et al. yielded the highest volume of delipidated plasma, was simple, and was consistently effective. Delipidated plasma retained full BChE activity and retained its native tetramer structure. BChE in delipidated plasma was efficiently captured by immobilized monoclonal antibodies B2 18-5 and mAb2. These monoclonals recognize conformational epitopes on native BChE and do not bind completely denatured BChE (Peng et al., 2015, 2016). This means the delipidating agents, dextran sulfate and Aerosil 380F, did not denature BChE.

Plasma was visibly clear after it was delipidated with dextran sulfate/CaCl_2_, centrifuged, and allowed to settle. However, when 100 mL of this plasma was pumped onto a 2 mL Hupresin affinity column, a layer of dextran particles accumulated on the surface of the Hupresin column. This raised the concern that the particles could change the properties of our chromatography media when we process liters of plasma. We solved this potential problem by filtering the delipidated plasma through a 0.8 micron filter. The filtered plasma left no dextran particles on the affinity column. It was concluded that delipidated plasma will require filtration before it is used for column chromatography to purify BChE from liters of plasma.

Delipidation is a critical step in purification of BChE from human plasma typically available from the Red Cross. The method of Masseyeff et al. (1965) with the attendant modification described in this paper provides a workable method for delipidation. The method may have application for delipidating milk containing recombinant human proteins (Huang et al., 2007; Wang et al., 2013).

## Author contributions

SO and OL performed experiments. OT contributed information on delipidation. SO, OT, and OL prepared the manuscript.

### Conflict of interest statement

The authors declare that the research was conducted in the absence of any commercial or financial relationships that could be construed as a potential conflict of interest.

## Abbreviations

BChE: butyrylcholinesterase (accession number P06276)
PAGE: polyacrylamide gel electrophoresis.
